# Genomic characterization of colitis-associated colorectal cancer

**DOI:** 10.1186/s12957-018-1428-0

**Published:** 2018-07-02

**Authors:** Hitoshi Kameyama, Masayuki Nagahashi, Yoshifumi Shimada, Yosuke Tajima, Hiroshi Ichikawa, Masato Nakano, Jun Sakata, Takashi Kobayashi, Sumana Narayanan, Kazuaki Takabe, Toshifumi Wakai

**Affiliations:** 1 Division of Digestive and General Surgery, Niigata University Graduate School of Medical and Dental Sciences, 1-757 Asahimachi-dori, Niigata, Niigata 951-8510 Japan; 2 Department of Surgical Oncology, Roswell Park Cancer Center Institute, Buffalo, NY USA; 3 Breast Surgery, Roswell Park Cancer Institute, Buffalo, NY USA; 4 Department of Surgery, University at Buffalo Jacobs School of Medicine and Biomedical Sciences, The State University of New York, Buffalo, NY USA

**Keywords:** Colitis-associated cancer, Colorectal cancer, Genomic characterization, Next-generation sequencing

## Abstract

**Background:**

Inflammatory bowel disease (IBD), which includes ulcerative colitis (UC) and Crohn’s disease (CD), is a chronic, idiopathic, repeated inflammatory disease. Colorectal cancer (CRC) that develops in patients with IBD is known as colitis-associated colorectal cancer (CAC), but the underlying carcinogenic mechanism remains unclear. Genomic analysis of sporadic CRC has been well described based on next-generation sequencing (NGS) data. Using NGS, we compared all exons of 415 cancer-associated genes in patients in Japan and the USA who had CRC and found similar genomic alteration patterns among the two populations. However, genomic analysis of CAC has not been thoroughly investigated.

**Main body:**

The molecular pathogenesis of CAC shares many features with sporadic CRC, but there are distinct variations in the time and frequency of some alterations. Gene alterations in CAC are gradually being elucidated using genomic sequencing analyses. Some studies have shown that gene alteration patterns differ between UC and CD. The carcinogenesis of CAC depends on unique environmental, genetic, and immunological factors.

**Conclusions:**

In this review, we have discussed the differences in genomic alterations between sporadic CRC and CAC. NGS in patients with IBD has the potential to detect early CAC and to suggest therapeutic targets.

## Background

Inflammatory bowel disease (IBD) is a chronic, idiopathic, repeated inflammatory disease that includes ulcerative colitis (UC) and Crohn’s disease (CD) [1]. IBD has distinct pathological and clinical characteristics; however, its etiology has not been completely elucidated [2].

IBD with colonic involvement markedly increases the risk of colorectal cancer (CRC). However, the detection of dysplastic or early malignant lesions on colonoscopy in patients with UC can be difficult, thus making the investigation of colitis-associated cancer (CAC) challenging.

Recently, the genomic analysis of sporadic CRC has been well-demonstrated using whole-exome sequencing or clinical target sequencing by next-generation sequencing (NGS) technology. Although these analyses have not been thoroughly utilized for analyzing CAC, limited studies have demonstrated that the mutations in CAC are similar to those in sporadic CRC. On the contrary, the timing of acquisition of mutations in particular genes may differ between CAC and sporadic CRC. This review focused on the results of genomic analysis of CAC, particularly on the differences in genomic alterations between sporadic CRC and CAC.

This study was approved by the ethics committee of the Niigata University Medical and Dental Hospital (No. 772).

## Ulcerative colitis

UC, which is a form of IBD which affects the mucosal layer of the colon, has become increasingly prevalent worldwide [1]. Recently, the prevalence of UC has been increasing worldwide, with the highest rates in Europe (505 per 100,000), Canada (248 per 100,000), and the USA (241 per 100,000) [1]. In Japan, the prevalence rate of UC was 121.9 per 100,000 in 2013 [3]. UC is characterized by relapsing and remitting colonic mucosal inflammation. The therapy for UC aims to resolve clinical symptoms, such as rectal bleeding and diarrhea [4] and to induce and maintain endoscopically confirmed remission, with the long-term goal of preventing disability and CAC [1].

## Crohn’s disease

CD is an inflammatory disease that affects the entire digestive tract from the mouth to the anus [5]. Its pathogenesis is not completely understood, but it is thought to develop due to environmental triggers in genetically susceptible patients [6]. The incidence of CD is also increasing worldwide. Kalla et al. reported that the incidence of CD was 29.3 per 100,000 in Australia, 20.2 per 100,000 in Canada, and 10.6 per 100,000 in northern Europe [6]. In Japan, the number of patients with CD has markedly increased. The prevalence rate in Japan was 2.9 per 100,000 in 1986 and increased to 13.5 per 100,000 by 1998 [7]. Patients with active CD often have poor quality of life which is secondary to abdominal pain, bowel obstruction, and fistula. Therapeutic agents for CD aim to treat or prevent these complications. Therefore, it is very important to ensure the best outcomes using a multidisciplinary approach toward treatment [6].

## Epidemiology of colitis-associated cancer

CAC was first recognized as a complication of UC by Crohn and Rosenberg in 1925 [8, 9], with malignancy developing in the colon or rectum in areas with active inflammation. It is listed as the cause of death in 10–15% of all patients with IBD [10–13]. The main risk factors for CAC include onset of IBD in young age, family history of sporadic CRC, longer duration of IBD, greater extent of colitis, existence of primary sclerosing cholangitis, increased severity of colitis, pseudopolyps, and dysplasia [14], whereas protective factors include use of folic acid, ursodeoxycholic acid, and 5-ASA, total proctocolectomy, and compliance with CRC surveillance (Table 1). The potential risk of CAC in sufferers with IBD is 1.5–2 times greater than that in the general population [14–16].

**Table 1:** Risk and protective factors for CAC patients

Risk factors	Protective factors
Young age onset	Folic acid use
Family history of sporadic CRC	UDCA use (in patients with PSC)
Long duration of IBD	5-ASA treatment
Increased extent of colitis	Colectomy
PSC	Compliance with CRC surveillance
Severity of colitis	
Pseudopolyps	
Dysplasia in UC	

*CAC* colitis-associated cancer, *CRC* colorectal cancer, *UDCA* ursodeoxycholic acid, *IBD* inflammatory bowel disease, 5-*ASA* 5-aminosalicylic acid, *PSC* primary sclerosing cholangitis, *UC* ulcerative colitis

CAC in patients with UC only accounts for 1% of all CRC cases [10]. However, it is a serious sequela of the disease and accounts for one sixth of all deaths in patients with UC [17]. There is a consensus that the risk of UC-associated cancer is highest in patients with extensive disease for a long duration [9]. Teenagers with pancolitis have a lifetime risk of CRC that exceeds 15% [14]. Fumery et al. reported that, in patients with UC with low-grade dysplasia, the pooled annual incidence of CRC was 0.8% and that of advanced neoplasia was 1.8% [18]. The high-risk features associated with dysplasia progressing to CAC in patients with UC include concomitant primary sclerosing cholangitis (odds ratio, 3.4; 95% confidence interval, 1.5–7.8), distal location (2.0; 1.1–3.7), and multifocal dysplasia (3.5; 1.5–8.5) [14]. Advanced-stage CAC in Japanese patients with UC has a worse prognosis than that of sporadic CRC. However, no difference was found in survival when CAC is at an early stage [19]. In Japan, the proportion of patients with UC who underwent surgery for CAC increased from 13.8% in 2008 to 20% in 2013 [20].

The risk of CRC in CD was reported to be 1.7–2.4 times greater than in the general population [6, 14]. Moreover, there exists a high risk of small intestine cancer in patients with CD [6]. In Japan, the incidence of CD-associated cancer has been on the rise, and the commonest site of occurrence is anorectal lesion. Sasaki et al. reported that the 5-year survival rate for CD-associated cancer was only 46.2%, compared with 89% in UC-associated cancer because of delayed diagnosis in patients with CD [21].

## Carcinogenesis

**Fig. 1:**
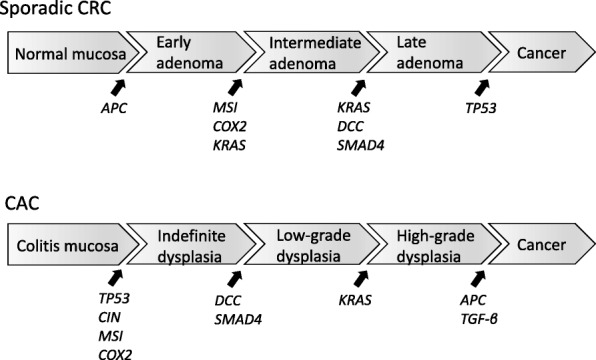
Differences in molecular pathogenesis of sporadic CRC and CAC. The loss of *APC* occurs early in the development of sporadic CRC, whereas it is usually a late event in CAC. *TP53* mutations appear early in CAC, even prior to the development of dysplasia, but late in sporadic CRC. *CRC* colorectal cancer, *CAC* colitis-associated cancer, *APC* adenomatous polyposis coli, *MSI* microsatellite instability, *COX2* cyclooxygenase 2, *KRAS* Kirsten rat sarcoma viral oncogene homolog, *DCC* deleted in colorectal carcinoma, *SMAD4* SMAD family member 4, *CIN* chromosomal instability, *TGF-β* transforming growth factor-β

Carcinogenesis of CAC involves transition from low- to high-grade dysplasia and comprises various gene alterations [12]. The molecular pathogenesis of CAC shares many features with sporadic CRC, but there are differences in the time and frequency of some alterations in the dysplasia–carcinoma sequence [14] (Fig. 1). As an illustration, the decline of the adenomatous polyposis coli gene takes place at the beginning with the development of sporadic CRC, although it is generally a delated event within the progression of UC-associated cancer. In addition, p53 mutations appear as important early events in CAC, even prior to the development of dysplasia, yet they occur late in the progression of sporadic CRC [22]. The loss of heterozygosity of p53 is correlated with malignant progression in UC. In a study by Burmer et al., it was detected in 6% of biopsy samples without dysplasia, 33% with low-grade dysplasia, 63% with high-grade dysplasia, and 85% with adenocarcinoma [23]. Furthermore, p53 mutations were found in areas of mucosal inflammation, indicating that chronic inflammation may be mutagenic [24]. On the other hand, the reported rate and timing of microsatellite instability (MSI) are similar in CAC and sporadic CRC [25, 26].

In IBD, the pathogenesis of CAC is related to chronic inflammatory changes with subsequent genetic alterations via mechanisms, such as the production of mucosal inflammatory mediators, oxidative stress, and changes in immune receptor expression on epithelial cells [12]. Moreover, it has been demonstrated in animal models that inflammatory mediators, such as IL-6, NF-κβ, and Stat3, play important roles in the progression of CAC [27]. Interestingly, a bioactive lipid mediator sphingosine-1-phosphate plays a pivotal role in the progression of CAC in a mouse model [28]. Further investigation is required to examine the roles of these mediators in human patients with CAC.

In UC, the colonic epithelium undergoes recurrent cycles of inflammation and tissue repair, resulting in the accumulation of reactive oxidative species which can cause further epithelial damage and lead to dysplasia [29]. The release of proinflammatory cytokines including NF-κβ is also a well-known mechanism [25]. The histopathogenesis of UC-associated CRC involves a step-wise progression from inflamed and hyperplastic epithelia to flat dysplasia and finally to adenocarcinoma [30].

Carcinogenesis of cancers associated with CD remains unclear compared with that of UC [31]. In CD, dysplasia occurs more often in areas close to other primary tumors [22]. Sigel et al. found that dysplasia was found adjacent to a carcinoma in 87% of cases and distant in 41% of cases [32]. Moreover, NF-κβ is thought to be associated with the pathogenic mechanism of CD [33].

## Genomic alterations in sporadic colorectal cancer

CRC is the third most common cancer worldwide [34]. In the USA, it is the second most common cause of cancer-related deaths [35]. Also, in Japan, the incidence of CRC has increased dramatically in the last decade. CRC is the leading cause of death in females, and the third leading cause of death in males among malignant neoplasms [19]. The well-known risk factors for CRC include male sex, older age, personal history of CRC, high body mass index, and lower activity [14]. Sporadic CRC typically develops from a premalignant adenoma through mutations in genes, such as *APC*, *KRAS*, *DCC*, and *TP53* (Fig. 1). The Cancer Genome Atlas (TCGA) Network reported the results of whole-genome sequencing for 224 patients with CRC, with 16% found to be hypermutated [36] and 84% to be non-hypermutated. Excluding the hypermutated cancers, 24 genes were significantly mutated, of which the eight most frequently reported were *APC*, *TP53*, *KRAS*, *PIK3CA*, *FBXW7*, *SMAD4*, *TCF7L2*, and *NRAS*. Other altered pathways in CRC may include mutations in the PI3K and RAS-MAPK pathways, deregulation of TGF-β signaling, and changes in WNT signaling pathway, which occurred in 93% of all tumors [36]. TCGA data showed that non-hypermutated CRCs cannot be distinguished at the genomic level; on the other hand, right-sided CRCs were more likely to be hypermethylated than those of CRCs at other sites.

## Genomic alterations in Japanese patients with colorectal cancer

Although ethnic and geographical differences may exist in the genomic alterations in CRC, most genomic data has been obtained from western countries, with data lacking from Asian countries. Recently, we investigated all exons of 415 cancer-associated genes, including major driver genes, in patients with CRC from Japan and the USA using NGS to compare the western and Asian populations [37]. After data analysis, we correlated the mutation burden with DNA mismatch repair status, obtained clear genomic mutational signatures, and identified genomic alteration patterns in CRC patients in Japan and the USA, which were similar to whole-exome sequencing data by TCGA.

We found hypermutated tumors in 8% of Japanese patients with CRC and 2% of American patients with CRC, both of which are commonly correlated with DNA mismatch repair deficiency (MMR-D) evaluated by immunohistochemical staining of MMR proteins, such as MLH1, MSH2, MSH6, and PMS2 [37]. We also identified overall similarities in the detection of actionable oncogenic driver genes in Japanese and American patients with CRC. For instance, genomic changes in oncogenic pathways such as the cell cycle, RAS/RAF, PI3K, and WNT were identical in Japanese and American patients; however, there are some distinct differences between the two populations. We found significant differences in *ERBB2*, *APC*, *TP53*, *CDKN2A*, and *NRAS* mutations between Japanese and American patients, which may show epidemiological distinctions between the two populations.

We further compared our results with the data obtained from TCGA [37] and found a difference in the *BRAF* mutation patterns between the two studies. In the TCGA-CRC cohorts, *BRAF* mutations were predominantly seen in the V600E hotspot, which is often restricted to hypermutated tumors. TCGA data showed that in non-hypermutated tumors, the rate of *BRAF* mutations of right-sided tumors were also significantly higher than that of tumors at sites. In contrast, our results showed that both Japanese and American patients with CRC had many types of non-V600E mutations inside and outside the kinase domain, including D594G, a kinase-dead BRAF that can drive tumor development through interactions with CRAF. Furthermore, we showed that *BRAF* mutations were recognized in both right- and left-sided tumors [37].

## Genomic alterations in colitis-associated carcinoma

Several previous reports have described the histopathological and genetic features as well as the roles of immune response and cytokines in CAC compared with sporadic CRC [12]. Kinugasa demonstrated that patients with high-grade dysplasia and those with CAC have increased β-catenin transcriptional activity that may contribute to increased claudin CL1 expression [2]. An association with MSI has been demonstrated in patients with UC, and the higher rate of MSI in long-standing UC is likely concerned with the genomic instability generated by chronic inflammatory stimulation [12]. Robles et al. reported whole-exome sequencing data, using human CAC samples [38]. Components of the Rho pathway that are responsible for cell motility and cytoskeleton remodeling include genes such as *RAC1*, *DOCK2*, *DOCK3*, *PREX2*, and *RADIL*, which were found to be frequently mutated in CAC compared with sporadic CRC. EP300 and TRRAP, which are epigenetic regulators and chromatin modifiers, were found to be more frequently mutated in CAC than in sporadic CRC. ERBB2, 3, and 4 pathways were found to be upregulated/amplified more often in CAC, but the c-MYC pathway was equally expressed in CAC and sporadic CRC [39]. Robles et al. also noted that, despite CAC and sporadic CRC having similar missense mutations within the DNA-binding domain of p53, the identity and molecular distribution of single substitution mutations were different [38]. Specifically, in patients with CAC, there were no mutations at the R273 hotspot, and just one mutation was discovered at each of the R248, G245, and R175 hotspots, which are more commonly mutated in patients with sporadic CRC. Conversely, patients with CAC had more mutations in the R282, R158, H179, and R342 hotspots, which are rarely identified in patients with sporadic CRC. They showed that the Rho and Rac pathways were affected in 10 patients with UC-associated cancer but in only 3 with CD-associated cancer (*p* = 0.025), indicating that these pathways may be preferentially activated in UC. WNT pathway genes were altered in almost all sporadic CRCs, most commonly *APC* [38]. Conversely, a lower rate of *APC* inactivation and higher rate of SOX9 transcription factor inactivating mutation were seen in the CAC cohort [38]. This indicates that CAC has a unique molecular profile which differs inherently from sporadic CRC, potentially providing clues to the etiology of CAC [38].

**Fig. 2:**
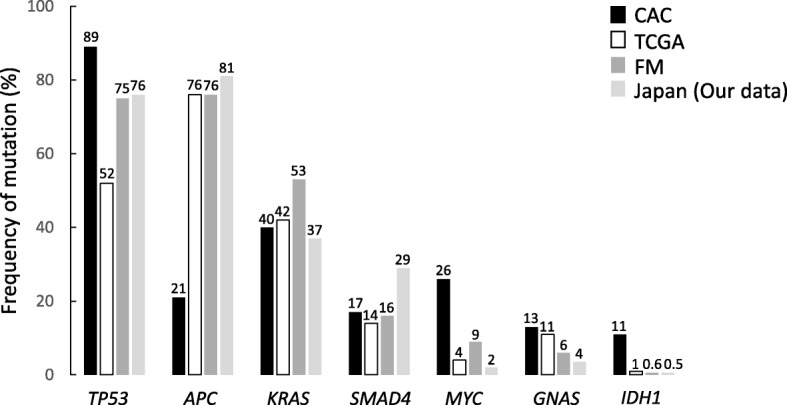
Comparison of the frequency of genetic alterations in CAC and sporadic CRC. *TP53* mutations are the most commonly occurring mutations in CAC. However, the mutations in *APC* are less frequent in CAC than in sporadic CRC. Genomic alteration patterns in Japanese and American patients are similar. *CAC* colitis-associated cancer, *TCGA* The Cancer Genome Atlas, *FM* Foundation Medicine, *APC* adenomatous polyposis coli, *KRAS* Kirsten rat sarcoma viral oncogene homolog, *SMAD4* SMAD family member 4, *IDH1* isocitrate dehydrogenase

**Fig. 3:**
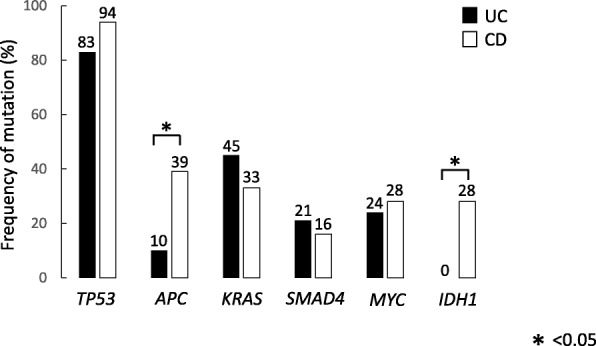
Comparison of the frequency of genetic alterations in UC and CD. The frequency of genetic alterations shares many features between UC and CD, but there are also some differences between the two. *TP53* mutations are the most commonly occurring mutations in UC and CD. *APC* and *IDH* alterations are significantly more frequent in CD than in UC. *UC* ulcerative colitis, *CD* Crohn’s disease, *APC* adenomatous polyposis coli, *KRAS* Kirsten rat sarcoma viral oncogene homolog, *SMAD4* SMAD family member 4

Yaeger analyzed genomic alterations in over 300 cancer-related genes in 47 CACs: 29 UC-associated and 18 CD-associated [40]. Using NGS analysis, they found 6.2 genomic alterations per tumor. Also, they showed that genomic alterations in *TP53*, *IDH1*, and *MYC* were significantly more frequent and mutations in APC were less frequent than those reported in sporadic CRCs in TCGA database. The frequency of gene alterations in CAC were *TP53* (89%), *APC* (21%), *KRAS* (40%), *SMAD4* (17%), *MYC* (26%), *GNAS* (13%), and *IDH* (11%) (Fig. 2). The activation of RTK/RAS signaling was common in CAC (UC, 57% and CD, 72%). WNT pathway components such as TGF-β and MYC were altered in approximately half of patients with CAC (UC, 41% and CD, 56%). *IDH* mutations in CAC were more common than in sporadic CRC. *APC* and *IDH* alterations were significantly more common in CD than in UC (Fig. 3). The specificity of *IDH* mutations in CD suggests its potential as a therapeutic target. The unique genetic mutations in CAC may also enable new diagnostic and screening tools to detect early CAC in patients with IBD [39].

## Conclusions

IBD is associated with an increased risk of CRC in which the carcinogenesis of CAC depends on unique environmental, genetic, and immunologic factors compared with sporadic CRC [41]. Although genomic sequencing for patients with IBD using NGS technology has not been thoroughly investigated, recent studies regarding CAC have suggested that there are several mutational differences between patients with sporadic CRC and those with CAC. Genomic sequencing for patients with IBD has the potential to identify specific genomic alterations in CAC which may lead to early detection and may identify potential molecular targets for the treatment of CAC.

## Abbreviations

CAC: Colorectal cancer
CD: Crohn’s disease
CRC: Colorectal cancer
IBD: Inflammatory bowel disease
MMR-D: Mismatch repair deficiency
MSI: Microsatellite instability
NGS: Next-generation sequencing
TCGA: The Cancer Genome Atlas
UC: Ulcerative colitis
